# Methoprene, a Juvenile Hormone Analog, Causes Winter Diapause Elimination in Univoltine Bee Species *Osmia bicornis* L.

**DOI:** 10.3390/ani13213344

**Published:** 2023-10-27

**Authors:** Karol Giejdasz, Monika Fliszkiewicz, Oskar Wasielewski

**Affiliations:** Department of Zoology, Poznań University of Life Sciences, Wojska Polskiego 71C, 60-625 Poznań, Poland; monika.fliszkiewicz@up.poznan.pl (M.F.); oskar.wasielewski@up.poznan.pl (O.W.)

**Keywords:** diapause termination, emergence, methoprene, overwintering, solitary bee

## Abstract

**Simple Summary:**

Diapause is physiological state of developmental arrest and dormancy, which is genetically determined and mediated by neurohormones. Diapause process is an opportunity for insects to survive seasonal environmental changes. Red mason bee *Osmia bicornis* is an univoltine species with imaginal diapause, and it is used for crop pollination. Our work shows that obligatory diapause in *O. bicornis* can be modified by a juvenile hormone analog. Treating pupae or young imagoes in cocoons during the pre-wintering period with a JHA (methoprene) can prevent winter diapause and induce the emergence of adult bees in October when development was accelerated in the laboratory or in November when bees developed outdoors according to their life cycle. More sensitive to methoprene is the imago than the pupa, but earlier application of JH analog can accelerate the emergence of adult bees. Our study suggests that JH levels during the pupal stage and soon after adult eclosion are related to hormonal regulation of winter diapause in *O. bicornis*. On the other hand, a better understanding of the mechanisms of reproductive diapause can help us improve the management of *Osmia* bees as commercial pollinators that can be used regardless of the emergence period in nature.

**Abstract:**

*Osmia bicornis* syn. *O. rufa* is a univoltine bee species in which adults fly in spring and the offspring overwinter as cocooned imagoes. The flight period of solitary bees is short, so methods of control for development and emergence time are needed to synchronize the activity of managed pollinators with blooming. In our study, we tested the effectiveness of a juvenile hormone analog for the prevention of winter diapause. Bees developed in settled nests outdoors or in the laboratory (22 °C) until the end of the pre-pupa stage, then cocoons were removed from the nest cells and treated with a JH analog—methoprene—during the pupa and young imago stages. Then, bees were activated at 25 °C until the adults left the cocoons. Topical application of methoprene to the cocoon at the pupa or imago stage induced the emergence of some adult bees in the pre-diapause period, while no adults emerged when the bees were not treated with methoprene. Most adults emerged (about 50%) when treated with methoprene on 3-week-old cocooned imagoes. Bees treated in the pupal stage had a lower emergence rate (20–30%), but adult bees emerged earlier. The emergence time of adults for the laboratory group was, on average, from 70 to 91 days, and that for outdoor groups was from 57 to 72 days.

## 1. Introduction

Diapause is a well-studied seasonal survival strategy and is influenced by several factors, such as species-specific ecological interactions, biogeography, life history and the physiology of many insects [1]. Biologically, the diapause phenomenon is defined as a dynamic state of low metabolic activity that is genetically determined and mediated by neurohormones that phenotypically affect individuals by decreasing morphogenesis, blocking reproduction and metamorphosis and increasing tolerance to extreme environmental conditions [2]. The diapause process is an excellent chance for insects to survive a great deal of seasonal changes in the environment [3]. Insects programmed to diapause pass through successive phases, including pre-diapause induction and preparation, the initiation of diapause, continuing maintenance, the termination of diapause and post-diapause physiology and behavior [4]. The pre-diapause program typically begins well before environmental conditions become adverse for further development or survival. The initiation of diapause may or may not be easy to determine. Many times, it is characterized by some metabolic combination (but not always), such as regulating energetic resources and hormone levels, e.g., prothoracicotropic hormone, ecdysone or juvenile hormone [5]. During the maintenance period, the organism is unresponsive to changes in the environment. This is usually characterized by decreases in metabolism, allowing organisms to conserve energy reserves. Diapausing insects gradually become more sensitive to diapause-terminating conditions as diapause intensity decreases. Termination usually depends on the reception of impulses concerning the environment and is characterized by resuming normal growth, development and activity in response to favorable environmental conditions (temperature, photoperiod) [1,5].

Depending on the species, diapause can occur at different stages, such as the embryonic, larval, pupal or adult stages. Reproductive diapause is a physiological adaptation that has evolved in some insects to survive adverse environmental conditions as an adult and is common in species of *Coleoptera*, *Lepidoptera*, *Diptera*, *Heteroptera*, *Orthoptera*, *Neuroptera* and *Trichoptera*. Female insects in reproductive diapause usually exhibit undeveloped ovaries, a build-up of fat body tissue, a reduction in accessory gland size and a change in metabolism and behavior [6,7]. There are a number of environmental factors that can play a crucial role in the completion of diapauses, such as temperature, photoperiod, light and water, and moisture. The shortening of the day-length in autumn or the change from long- to short-day photoperiods coupled with the lowering in temperature is sufficient to induce adult diapause in many insects [8,9], including some *Osmia* species [10,11,12]. Beyond the modifications of inducing factors, photoperiod and temperature, diapause is controlled by neuroendocrine processes [5,13,14]. Endocrine regulation is important, especially in reproductive diapause (i.e., a resting state with reduced metabolic activity), where juvenile hormone (JH) titers appear to play a crucial role [15]. A decrease in JH production in the *corpus allatum* (CA) induces the cessation of reproduction, specifically the arrest of vitellogenesis and regression of the ovaries [6].

**Figure 1:**
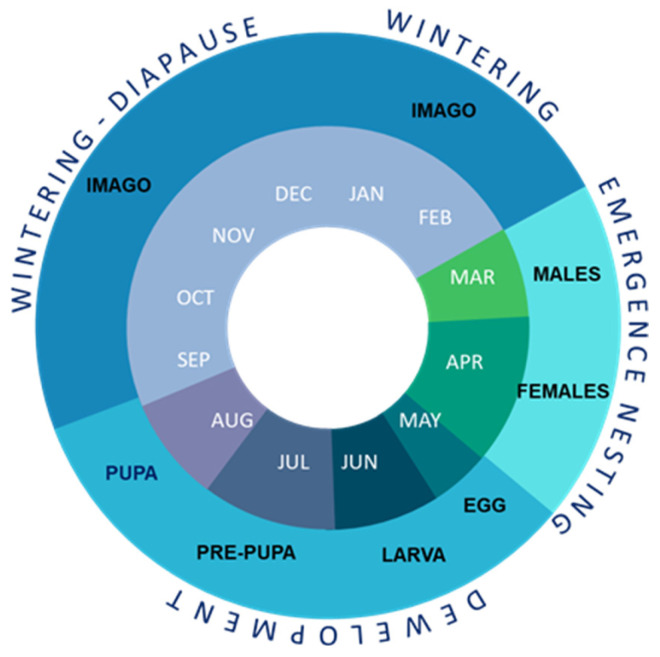
Life cycle of *Osmia bicornis* L.

The red mason bee, *Osmia bicornis* L., is a native European solitary bee showing promise as a manageable pollinator of fruit, especially apples, pears, plums, raspberries and strawberries [16]. This univoltine species completes its development from an egg to the imago stage in the spring and in the summer season [16,17]. Giejdasz and Wilkaniec [16] determined that under natural conditions and an average temperature of 14 °C, eggs matured in 4–9 days. More than one day after a larva hatches, it starts to eat pollen. *O. bicornis* have five larval instars. On average, a female larva feeds for 35.5 days, while the male larvae feeds for 31.6 days [16]. Next, larvae spin four-layered cocoons, which take between 3 and 6 days to complete, and then during the hot summer days it remains as a pre-pupae. Healthy pupae develop within cocoons into adults in August/September. Under natural conditions, normal pre-pupal and pupal stages have taken, together, 54.2 days for females and 52.6 for males. The duration of all of the stages takes approximately 100.8 days for females and 95.5 for males under natural conditions [16] (Figure 1). Adults eclose in September and overwinter in the imago stage inside a cocoon through the winter months [16]. Based on the classification of the eco-physiological phases of insect diapause [4], *O. bicornis* enters diapause in November and diapause termination occurs at the end of January [18,19]. For the next three months, the adults are still cocooned. This time corresponds to a post-diapause quiescence (February–April), during which different metabolic rates are observed depending on tissue (fat body or hemolymph) [11,20]. In natural conditions, as in other species of *Osmia*, the bees emerge the subsequent spring as the temperature rises [21]. The photoperiod has not been implicated in the diapause of *Osmia* species since development from egg to adult in these species takes place inside a sealed nest in complete darkness, but the use of higher temperatures at the end of diapause and post-diapause have been reported to induce the emergence of bees [22].

Most of the annual life cycle of *O. bicornis* is spent on development and overwintering, while the flight period of adult bees is short, so methods of control development and emergence time are needed to synchronize bee activity with blooming. The application of higher temperatures at the end of diapause and post-diapause may cause bees to emerge. This process proceeds slowly, which makes flights and blooming difficult to synchronize. Treating bees with temperature only does not prevent diapause or terminate intensive diapause. In addition to temperature, we need to apply an additional hormonal factor that could accelerate the end of diapause and allow for better synchronization for flights and blooming. Therefore, in the present study, the effect of the exogenous application of a JH analog, methoprene, as a factor that stimulates the end of adult diapause, bee activation and emergence is examined. The use of a JH analog for diapause control significantly increases the possibility of using red mason bees outside the flight period, and fruit and seed growers will have access to managed pollinators at various times, especially when crops are grown under cover.

## 2. Materials and Methods

The experiments were conducted in the summer and autumn of 2021 when the *Osmia bicornis* were in the pupal and pre-wintering periods (Figure 1). The experimental material consisted of pupae and young adult bees reared in artificial nests made of reed stalks, following the method of Wójtowski and Wilkaniec [23], and originated from nests settled by females in natural conditions in the botanical garden near the Department of Zoology (Poznań University of Life Sciences, Poznań, Poland). 

**Figure 2:**
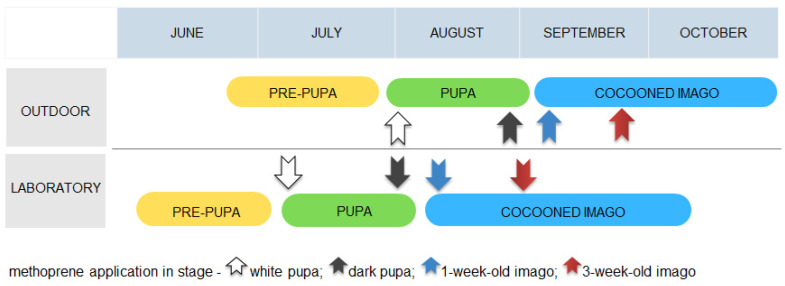
Application time of methoprene concerning bee stages and developmental conditions.

After the nesting period, the occupied nests were inspected, and the bees were divided into two groups. One was left outdoors at the nesting site (*n* = 317). The second was moved to the laboratory, where the insects (larvae, pre-pupa, pupa) developed faster at 22 °C (*n* = 480). The temperature outside during larval development ranged from 7.5 to 33.2 °C. The different stages of bees were obtained from occupied nests and transported to the laboratory according to the life cycle schedule (Figure 1). The experiment started when the bees reached pupation (for bees developing in nature, on the 7th of August, and for the laboratory group, on the 12th of July). The experiment was continued as the development of the bees progressed (Figure 2). The nest tubes were dismantled and the cocoons were removed from the nest cells when the bees were in the appropriate experimental stages of white pupa (outdoors: *n* = 80; laboratory: *n* = 120), dark pupa (outdoors: *n* = 80; laboratory: *n* = 120), 1-week-old imago (outdoors: *n* = 78; laboratory: *n* = 120) and 3-week-old imago (outdoors: *n* = 79; laboratory: *n* = 120). The individuals in one nest are generally of similar ages, so one cocoon from the nest was dissected to control the stage of bees, but this bee was not included in the sample. The pupa age was determined on the basis of the color. The youngest pupae, 1–3 days old, are all white (including eyes). At 22 °C, about 3 weeks later and in nature a month later, the pupae are all pigmented. After this time, the cocoons were dissected to confirm imago eclosion. Ten cocoons were controlled every day for a week, and when all of them included an imago, the experiment with the imago was started. Cocoons were sexed according to their size and position within the nest. Three groups (treated, vehicle control, negative control) of bees were selected separately for each developmental stage and condition (in nature or laboratory).

The experimental cocooned females were treated topically on the cocoon surface once a day for five consecutive days with 200 µg of methoprene dissolved in 5 µL acetone (Sigma, Sosnowiec, Poland) and cocooned males with 120 µg of methoprene dissolved in 3 µL acetone [24,25,26]. Appropriate volume and concentration of methoprene were prepared according to our previous experience and experiments and based on authors who performed their experiments on *Apis mellifera*, a honeybee who belongs, similar to *Osmia*, to the *Apoidea* superfamily and has similar body weight [18,27,28]. The vehicle control was treated with 5 µL of acetone (female) or 3 µL (male) and the negative control was not treated. The bee cocoons in the experimental group and the two control groups were kept under the same conditions. During application time, the cocoons of all groups were kept separately on plates with specially prepared holes and placed in a climate chamber (model MLR-350H Sanyo) at a temperature of 22 °C ± 0.5 °C and relative humidity of 60% ± 1%. After the last application, the cocoon storing temperature was 25 °C ± 0.5 °C and, under these conditions, bees were kept until emergence. Methoprene as a juvenile hormone (JH) analog was chosen because JH has been implicated in the regulation of reproductive diapause. This hormone is required by many insects to stimulate reproductive tissue and egg development. The JH analog, methoprene, was kindly provided by Prof. Dalibor Kodrik (Institute of Entomology, Ceske Budejovice, Czech Republic).

The number of emerged *O. bicornis* adults was scored each day until live bees had emerged from the cocoons. Adult males and females were identified according to morphological features to confirm the sex of the bees. These data were used to estimate the value of the bee emergence rate, which was expressed as percentages of emerged adult bees (males and females) to the total number of cocooned bees.

After the experiment was finished, cocoons with non-emerged bees were dissected and the developmental stages of dead bees were determined. Moreover, it was checked whether there was a parasitic infection. 

The distribution of the emergence time variable was tested with the Shapiro–Wilk test and the lack of a normal distribution was confirmed. The Kruskal–Wallis test (one-way ANOVA by ranks) and multiple pairwise comparisons with Bonferroni correction were used to compare the emergence times of bees at various developmental stages (white pupa, dark pupa, 1-week-old imago, 3-week-old imago) and separately for developmental conditions. The time of emergence of individuals for all experimental groups was measured from the last date of application of methoprene to 3-week-old imago, separately for the outdoor and laboratory groups.

Relationships between the bee stage treated with JH analog and the percentage of emerged adults were determined using the chi-squared test for bees developed in nature and laboratory, separately. To perform the test, a cross table of observed and theoretical values was created which contained the number of emerged bees induced by methoprene treatments in various stages and the number of non-emerged bees which remained in cocoons. All analyses were performed at a significance level of α = 0.05, separately for males and females and the developmental conditions, using the software Statistica v. 13.1.

## 3. Results

### 3.1. Bee Emergence Rate 

**Figure 3:**
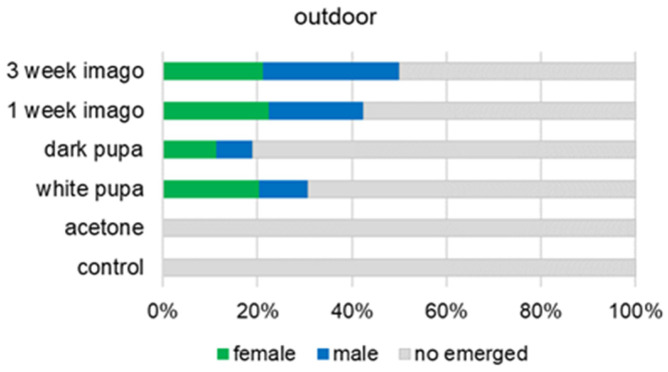
Percentage of adult bees emerged as an effect of methoprene treatment at different stages. The larval development of the bees proceeded outdoors. In the control and acetone groups, methoprene was not used. Chi-square test: χ^2^ = 12.35, df = 3, *p* = 0.006.

**Figure 4:**
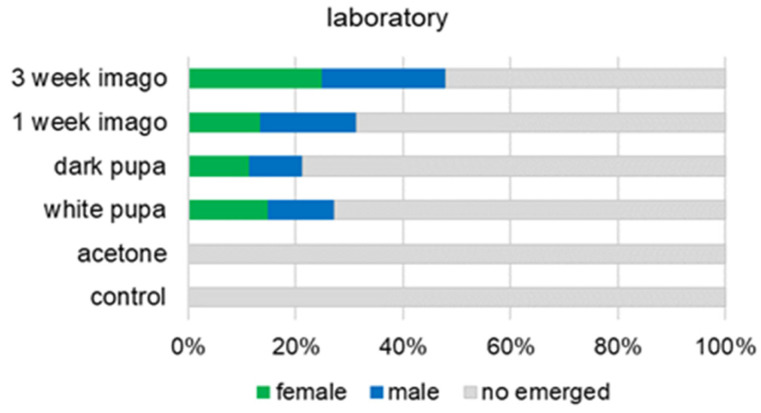
Percentage of adult bees emerged as an effect of methoprene treatment at different stages. The development of the bees proceeded in the laboratory. In the control group, methoprene was not used. Chi-square test: χ^2^ = 9.83, df = 3, *p* = 0.020.

Topical application of methoprene to the cocoon at the pupa or imago stage induced the emergence of some adult bees in the pre-diapause period. No adults emerged in the control group (acetone and intact), in which the bees were not treated with methoprene (Figure 3 and Figure 4). The frequency of adult bee emergence is related to the time of methoprene application (outdoor: χ^2^ = 12.35, df = 3, *p* = 0.006; laboratory: χ^2^ = 9.83, df = 3, *p* = 0.020). Regardless of the developmental conditions, the number of adults emerged the least when methoprene was applied at the dark pupal stage (18.99%—in outdoor group) (Figure 3). The most sensitive developmental stage to methoprene application was the 3-week-old imago (Figure 3 and Figure 4). In the outdoor group of bees, we noted a 50% emergence rate (male—28.75%; female—21.25%) and, similarly, in the laboratory group this was 47.86% (male—22.86%; female—25%). 

### 3.2. Bee Emergence Time

**Figure 5:**
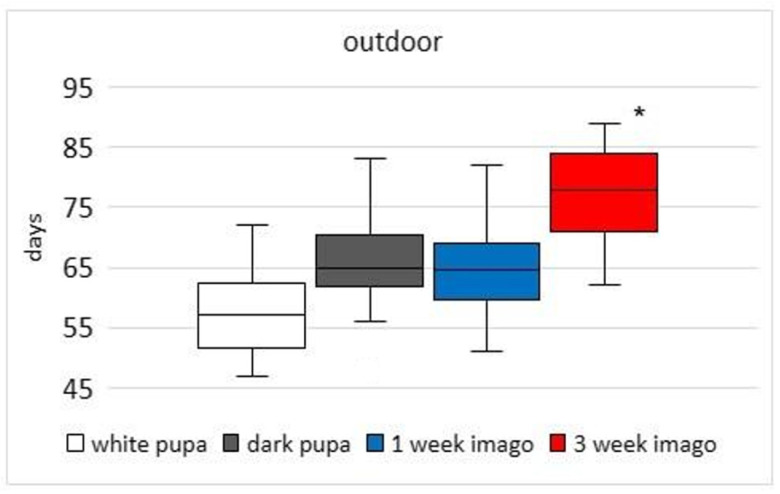
Comparison of the emergence time (days) of adult bees treated with methoprene at different stages. The larval development of the bees proceeded outdoors. The emergence time of adults was measured from the last date of methoprene application (20.09). The boundaries of the boxes indicate the 25th and 75th percentiles; the line within the box marks the median. Whisker boundaries indicate minimum and maximum values. * indicates statistically significant differences between groups.

**Figure 6:**
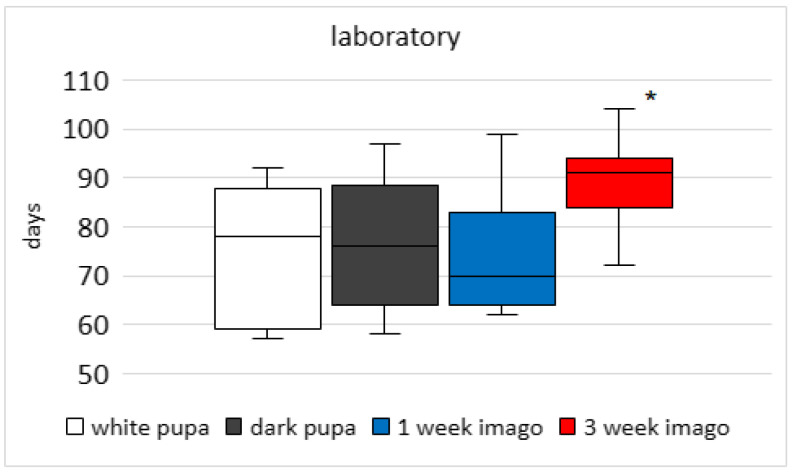
Comparison of the emergence time (days) of adult bees treated with methoprene at different stages. The development of the bees proceeded in the laboratory. The emergence time of adults was measured from the last date of methoprene application (22.08). The boundaries of the boxes indicate the 25th and 75th percentiles; the line within the box marks the median. Whisker boundaries indicate minimum and maximum values. * indicates statistically significant differences between groups.

The emergence time of adults for all groups was measured from the last date of methoprene application (20.09 for the outdoor group; 22.08 for the laboratory group). Bees after methoprene treatment emerged faster when larval development occurred outdoors than in the laboratory. Throughout the experiment (until the last bees appeared), in the acetone and intact groups the emergence of bees was not noticed. The time of methoprene application in selected developmental stages has effects on the time the imago remains in the cocoon (outdoor K-W test H (3; 120) = 58.47; *p* < 0.001; Figure 5; laboratory K-W test H (3; 155) = 44.64; *p* < 0.001; Figure 6). Average median emergence times of the outdoor bee groups were between 57 and 78 days (dependent on developmental stage) compared with between 70 and 91 days in laboratory groups (Figure 5 and Figure 6). Multiple comparison tests showed that the medians of the 3-week-imago group were higher than those in the other groups under outdoor and laboratory conditions. Statistical differences were not observed between white pupa, dark pupa, and 1-week-old imago stages. In the outdoor bee groups, the first imago emerged after 47 days and the last 72 days after methoprene application (for white pupa, developmental stage) (Figure 5) and stayed in the cocoon for 25 days. For comparison, in the laboratory group the first imago emerged after 72 days and the last 104 days after application (for 3-week-old imago stage) (Figure 6). 

**Figure 7:**
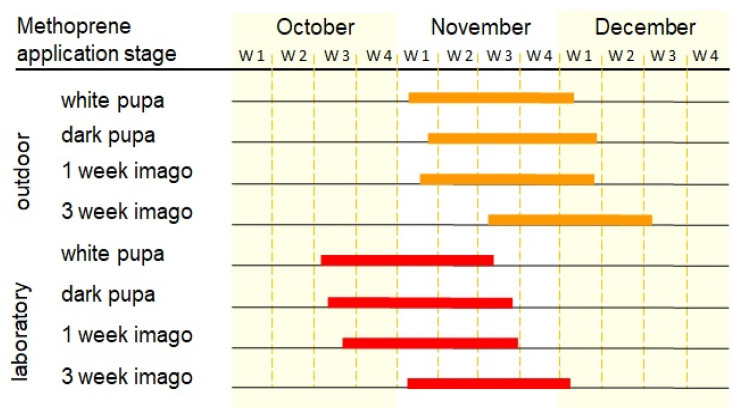
Duration and schedule of emergence of adult bees treated with methoprene. The beginning of the bar is determined by the first emerged adult and the end by the last.

All adult bees emerged after methoprene application in the months from October to December (Figure 7) (under natural conditions, adults will emerge next spring in April). Adults from the outdoor group started leaving the cocoons in early November and finished in mid-December. The emergence period of adults from the lab group was shifted about two weeks early.

### 3.3. Bee Mortality in the Cocoon

The cocoon dissection showed that the bees died mostly in the imago stage (90.8–97.5% of all non-emerged cocoons), and only a low percentage died in the pupa stage or were infected with a parasite (0–3.1%). In the group of bees treated with methoprene at the white pupae stage, 5.5% and 4.2% of white pupae were dead; 3.7% and 3.5% of dark pupae were dead in the outdoor and laboratory groups, respectively. Bee cocoons treated at the dark pupae stage contained 1.5% and 1.7% dead white pupae and 1.5% and 1.3% dark pupae in the outdoor and laboratory groups, respectively. There were 1.5% and 3.2% pupae dead in the group of 1-week-old imagoes and 2.5% and 3.6% in the group of 3-week-old imagoes in the outdoor and laboratory groups, respectively. The control group had 3.8% and 2.9% dead white pupae; 2.5% and 3.1% dark pupae; and 3.8% and 2.7% parasitized bees in the outdoor and laboratory groups, respectively.

## 4. Discussion

In this study, it was found that the application of the juvenile hormone analog reduced the emergence time of adult *O. bicornis* bees during overwintering time. Experimentally treated bees emerged faster than control bees. We also observed that in females, the effect of methoprene is more prominent than that in males. Endocrine regulation is especially important in reproductive diapause (i.e., a resting state with reduced metabolic activity), where juvenile hormone (JH) titers appear to play a crucial role. A decrease in JH production in the corpus allatum (CA) induces the cessation of reproduction, specifically the arrest of vitellogenesis and regression of the ovaries [5,7]. Generally, the administration of juvenile hormone or its analog methoprene into adult insects can exert both positive and negative effects on development and reproduction [18,19]. Our results indicate that the JH analog, methoprene, may play an important, positive role in the termination of diapause. It is well illustrated by the acetone control and untreated females and males.

An obligatory winter diapause has been described in many insects that overwinter at different developmental stages (from eggs to imago). In these species, the period of wintering and cooling is required to terminate diapause and continue insect development [29]. Also, the obligatory summer diapause in adults can usually stop after an intense duration of diapause [30]. Bee species of the *Osmia* genus that are used as managed pollinators are univoltine insects with obligatory winter diapause, but the inducing and terminating factors of diapause are not determined [29]. 

In *Osmia* bees, the wintering period, during which cocooned adults are exposed to low temperatures, lasts about 150 days in temperate climates [31]. Thirty days of low temperature in the laboratory is not enough to terminate the diapause of these insects, and the survival rate of these adult bees is very low at 0–40% [32]. In many species, the minimum time of cold exposure, after which they can restart development, seems significant. However, little is currently known about the physiological processes and mechanisms that regulate the termination of diapause [2].

In our study, we eliminated low temperatures, a factor associated with diapause termination, to control the effect of the JH analog (methoprene) as a factor of diapause prevention. During the experiment, newly eclosed adults inside their cocoons remained at 22 °C and after the last methoprene application at 25 °C to activate the bees and emerge. This time corresponds to the pre-wintering period, which is mentioned as one of the life cycle phases of *Osmia* bees [31]. 

In the present study, prolonged pre-wintering in the laboratory did not eliminate diapause in *O. bicorins* in the control group (without methoprene), in that no adult bees emerged and all individuals died in cocoons. This proves that warm temperatures do not eliminate obligatory diapause. But, if the facultative imaginal diapause in *Dolycoris baccarum* is regulated by a photoperiod, high temperatures can modify the photoperiod response and prevent diapause; but the consequence is to delay the pre-oviposition period [33]. 

Juvenile hormones are involved in the regulation of insect diapause at different stages [5]. The effects of JH during larval diapause are not clear. Most researchers show that more JH is found in the hemolymph of larvae that enter diapause than in larvae that continue pupation [34,35]. Larval diapause includes an increase in JH in the fourth and fifth instar larvae [36]. Opposite to these findings, JH analog application stimulates the prothoracic glands of diapausing *Omphisa* larvae and terminates larval diapause [37]. Similarly, in the maternal regulation of diapause in *Nasonia vitripennis* wasps, high levels of JH in the hemolymph stimulate the female to produce offspring that do not diapause, while low JH produces diapausing larvae [38]. Juvenile hormones are involved in the process of the regulation of imaginary diapause, and low JH levels may be a factor of diapause induction in adult insects [5,39]. In the Colorado potato beetle, JH levels were higher in pupae that produced non-diapause images than in pupae that produced diapause images. Also, JH was higher in non-diapause than diapause adult beetles [40]. In the presented study, methoprene application was performed in either the pupa or imago stage, and better diapause elimination effectiveness was after imago treatment. This shows that the imago is more sensitive to the effects of methoprene, although the emergence of adult bees is more rapid after pupa treatment.

Compensation of a JH deficit via the use of a juvenile hormone analog has a potential effect on imaginal diapause. There are reports that topical application of methoprene in the function of a JH analog effectively terminated reproductive diapause in the overwintering fly *Melinda pussila* [41]. Also, autumn forms of the bivoltine butterfly *Polygonia c-aureum*, which are disposed to overwinter, terminate diapause after treatment with methoprene [42]. In the case of summer diapause in the beetle *Geleruca daurica*, the start of diapause could be postponed when treating adults with a JH analog in the pre-diapause stage but could not terminate diapause when JHA was used during diapause, and this suggests that obligatory diapause can usually be terminated after a long duration of diapause [40].

In most insects, imaginal diapause has been described as the suspension of reproduction. At the time of adult eclosion, ovarian and oocyte development is arrested; also, vitellogenin synthesis and absorption is inhibited [12,43,44]. During diapause in adult insects, JH synthesis in the corpora allata glands is reduced and JH levels are low, which is an important factor that induces reproductive diapause [5,39]. Conversely, in insects without or after diapause, JH levels were high and the corpora allata glands showed activity [45].

In our study, the methoprene application and the high temperature in the pre-wintering period induced some of the individuals to be active and emerge, and this was demonstrated by the adult bees biting and moving out of the cocoon. Methoprene as a factor in diapause prevention was found to be at most 50% effective. The application of a juvenile hormone analog significantly stimulated ovarian development and induced vitellogenesis in immature female insects in a diapause condition and resulted in the termination of diapause [39,46]. In the previous study, we proved that the use of methoprene like a JH analog stimulated ovary growth, was effective in the termination of *O. bicornis* wintering, and caused the emergence of adult bees in late December after a 50-day cooling period at 4 °C [12,28]. Similarly, methoprene application terminated reproductive diapause in the butterfly *Caloptilia fraxinella* only when adults were treated in the middle of diapause [7]. Also, in the beetle *Aulacophora nigripennis*, methoprene broke diapause and 75% of individuals proceeded to mating [47]. In our previous work, methoprene was applied after a small incision of the cocoon [18]. This procedure is risky. It creates a probable risk of damaging the imago and it dying inside the cocoon. Based on our experience [29,30] and information that the chemical properties of methoprene (JH analog) will allow it to penetrate the cocoon and cuticle, we decided to apply the methoprene on the cocoon surface. The effectiveness of methoprene penetration and activity was estimated with comparison to the vehicle (acetone) and intact (non-treated) controls.

In our experiment, the emergence time was extended (60–90 days) and a lot of the imagoes, which do not feed during this time, died in the cocoons. One of the mortality reasons was the depletion of the energetic reserves. The emergence time is in inverse proportion to the wintering time and was about 80 days when the cooling lasted only 30 days [32]. In Osmia bees, during the pre-wintering period, the body weight loss of imago in cocoons at 22 °C was very rapid and slowed down when transferred to 4 °C. Moreover, prolongation of this period significantly reduces the fat body and increases mortality during wintering [10]. In our study, the pre-wintering period was extended, and the temperature in the laboratory (22 °C) supported the loss of energetic reserves. In the laboratory, the high temperature during the acceleration of development could increase metabolism and increase the metabolic demand for energy, consequently reducing the accumulation of energy reserves in the fat body. When *O. bicornis* development was conducted at 22 °C, adult mortality increased to 24.5% [48].

The bees developed about three weeks earlier in the lab than outdoors. Eclosion occurred in mid-October in the lab and in early November outdoors. However, adult bees started to emerge after methoprene treatment about two weeks earlier in the lab than outdoors. This difference decreased because emergence times were longer in bees reared in the lab. Adult eclosion of *O. lignaria* reared at 22 °C occurred about two weeks earlier than in those reared outdoors [10]. The time of *O. bicornis* development, which takes about 100 days outdoors, is reduced to about 80 days at 25 °C [16].

Most of the dead bees in the cocoons were adults as a result of the prolonged pre-diapause stage at high temperatures. Cocoon dissection also showed that bees died at the pupal stage or were infected with parasites in a low percentage. In the present study, mortality in the pupal stage after methoprene application and in the control group were similar. This suggests that methoprene application at the pupal stage does not disturb their development. In other studies, *O. bicornis* mortality during development at various temperatures was highest (1.3–13%) until the larva was cocooned, and pupal mortality ranged from 0 to 3.8% [48].

The presented study suggests that JH levels during the pupal stage and soon after adult eclosion are related to hormonal regulation of winter diapause in *O. bicornis*. In addition, we proved that methoprene can eliminate diapause in some individuals. 

## 5. Conclusions

The contribution to knowledge of overwintering time, including the diapause period in *O. bicornis*, will improve the management of this bee for crop pollination. The necessity of synchronous adult emergence of pollinators with a blooming period requires the termination of diapause or continuation of the overwintering period under laboratory conditions. The red mason bee can be used as a managed pollinator in greenhouses and field plots outside its natural flight period. Finally, our results may be used to devise methods to control diapause in *O. bicornis*, which enables the activation of adult bees at various times.

## Data Availability

The data presented in this study are available on request from the corresponding author.
